# Fullterm pregnancy in umbilical hernia

**DOI:** 10.4314/pamj.v8i1.71053

**Published:** 2011-01-31

**Authors:** Damien Punguyire, Kenneth V Iserson, Stephen Apanga

**Affiliations:** 1 Kintampo Municipal Hospital Kintampo, Ghana Kintampo Health Research Centre Kintampo, Ghana; 2 Department of Emergency Medicine, The University of Arizona, Tucson, AZ, USA

**Keywords:** Pregnancy complications, umbilical hernia, ventral hernia, Cesarean section, grand multiparity

## Abstract

While umbilical hernias frequently occur during pregnancy, the few reported cases of uterine or fibroid incarceration in ventral hernias during pregnancy all involved incisional abdominal wall defects from prior laparotomies and Cesarean sections; none involved umbilical hernias. We discuss the case of a 42-year-old well-developed, well-nourished grand multiparous woman (G8P7) with a huge umbilical hernia containing a 38-week gravid uterus, as well as her management and the avoidance of known complications that have occurred in similar incisional hernia cases. Successful pregnancy outcomes can occur in cases of pregnancies in ventral hernias, even in resourcepoor settings that have Cesarean section capabilities.

## Background

While umbilical hernias frequently occur during pregnancy, the few reported cases of uterine or fibroid incarceration in ventral hernias during pregnancy all involved incisional abdominal wall defects from prior laparotomies and Cesarean sections; none involved umbilical hernias [1]. Given the reported risks of pregnancies in anterior wall defects, we present a case where no permanent ill effects, to either the baby or mother, resulted from a fullterm pregnancy in an umbilical hernia.

## Patient and case report

**Figure 1:**
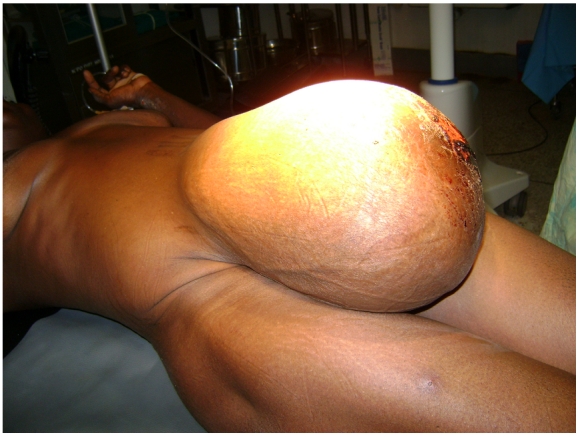
38-week gravid uterus in umbilical hernia-before surgery.

A 42-year-old well-developed, well-nourished grand multiparous woman (G8P7) with a huge umbilical hernia (Figure 1) presented to the outpatient clinic stating that she was in labor. She had vaginally delivered her previous children at home with the assistance of a traditional birth attendant. All were healthy. She had never had surgery.

On examination, she had normal vital signs and an unremarkable heart and lung exam. Abdominal examination revealed a huge protruding mass through the umbilical region with a 5-cm X 5-cm ulceration of the overlying skin. The uterus could be felt subcutaneously with a complete lack of overlying abdominal wall fascia and musculature. The fetus lay in a longitudinal position with the fetal head at the uterine fundus in the most dependent part of the umbilical hernia. Laboratory examinations were normal. Ultrasonography showed a full-term (38 weeks, 1 day) fetus with a normal heart rate, no gross congenital abnormalities, and the placenta in the upper uterine segment. Since uterine contractions were observed, the patient was prepared for emergency surgery.

**Figure 2:**
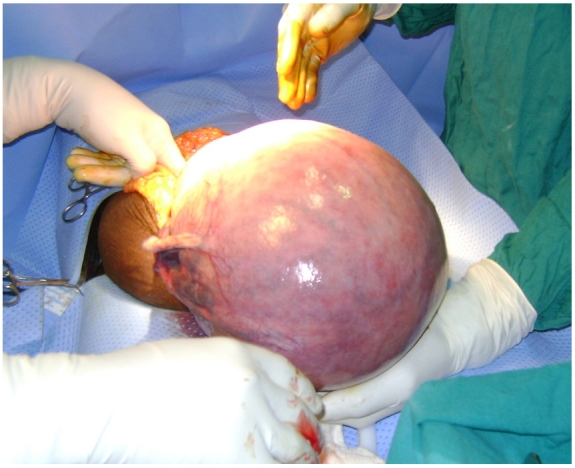
Full term pregnancy in umbilical hernia - uterus exposed during surgery.

Under general anesthesia, the skin over the umbilical mass was incised. This exposed the subcutaneous uterus (Figure 2). The redundant skin over the uterus, including the ulcerated skin, was excised and the uterus entered through the posterior-lower segment. The baby was delivered (Apgar 8/10; 9/10) and hemostasis achieved. After performing a bilateral tubal ligation, the uterus was replaced in its normal pelvic position; the widely separated fascia was dissected and reapproximated. The skin was closed primarily.

## Discussion

Umbilical hernias (protrusions of ≥5 mm and diameters of ≥10 mm from the abdominal skin surface) are present in about 15% of pregnant West African women [2]. The rate for Cesarean sections is less than 5% in most of sub-Saharan Africa [3]. Yet, while some reports exist of fibroids and gravid uteri in incisional hernias resulting from C-sections or laparotomies, there has been no prior report of a gravid uterus in an umbilical hernia.

Finding a gravid uterus in an anterior abdominal wall hernia is rare, and is usually found, as in this case, in multiparous patients [4,5]. The presentation and course of this pregnancy within an umbilical hernia was similar to prior reported cases of pregnancies in incisional hernias, albeit with minimal complications.

**Complications**

Multiple complications have been reported in association with pregnancies in anterior abdominal wall defects. The only previously reported complication that occurred in our patient was excessive stretching of the skin, causing ulceration due to friction between the hernia sac and other parts of the patient’s body and clothing.

Other reported complications, many of which can threaten the mother or fetus, include incarceration, miscarriage, premature labor, intrauterine hemorrhage, intrauterine growth retardation, intrauterine death, rupture of the lower uterine segment, ruptured abdominal wall, and death [5].

**Herniorrhaphy during C-Section**

It has been suggested that the laxity of the abdominal wall and the presence of an enlarged, hypertrophied uterus could weaken a repair. Despite these theoretical concerns, herniorrhaphy has been successfully performed as part of the Caesarean section with no increase in wound infection rates and no recurrences [6]. As in some previous cases, we chose to repair the hernia during the Caesarean section, since we could excise the redundant skin surrounding the pressure ulcer. Even though the recurrence rate after simple repair of anterior abdominal wall hernias is more than twice the rate when mesh is used, we did not use mesh in our repair, since it was unavailable at that time in our hospital [1].

**Follow-up**

Our patient’s repair was intact at follow-up >1 year after surgery; the baby was healthy and developing normally.

## Conclusion

Advanced pregnancies developing in ventral hernias can pose multiple risks. Yet even in resource-poor settings, successful pregnancy outcomes can occur in such cases, including the rare situation of a pregnancy in an umbilical hernia, if Cesarean section capabilities exist.

## Competing interests

There are no financial or competing interests associated with this manuscript. This manuscript has not been submitted elsewhere.

## Authors’ contributions

All authors participated in vital aspects of producing this article and approve the final version for publication.
